# Seroprevalence of Antibodies against Pkn1, a Novel Potential Immunogen, in *Chlamydia trachomatis*-Infected *Macaca nemestrina* and Human Patients

**DOI:** 10.1155/2014/245483

**Published:** 2014-06-18

**Authors:** Achchhe L. Patel, Prashant K. Mishra, Divya Sachdev, Uma Chaudhary, Dorothy L. Patton, Daman Saluja

**Affiliations:** ^1^Dr BR Ambedkar Center for Biomedical Research, University of Delhi, Delhi 110007, India; ^2^Bhaskaracharya College of Applied Sciences, University of Delhi, Delhi 110075, India; ^3^University of Washington, Seattle, WA 98195-6460, USA

## Abstract

*Chlamydia trachomatis* (CT) is an important cause of sexually transmitted genital tract infections (STIs) and trachoma. Despite major research into chlamydial pathogenesis and host immune responses, immunoprotection has been hampered by the incomplete understanding of protective immunity in the genital tract. Characterized vaccine candidates have shown variable efficacy ranging from no protection to partial protection *in vivo*. It is therefore a research priority to identify novel chlamydial antigens that may elicit protective immune responses against CT infection. In the present study we assessed the seroprevalence of antibodies against protein kinase1 (Pkn1), DNA ligaseA (LigA), and major outer membrane protein A (OmpA) following natural CT infection in humans and in experimentally induced CT infection in *Macaca nemestrina*. Antigenic stretches of Pkn1, LigA, and OmpA were identified using bioinformatic tools. *Pkn1*, *LigA*, and *OmpA* genes were cloned in bacterial expression vector and purified by affinity chromatography. Our results demonstrate significantly high seroprevalence of antibodies against purified Pkn1 and OmpA in sera obtained from the macaque animal model and human patients infected with CT. In contrast no significant seroreactivity was observed for LigA. The seroprevalence of antibodies against Pkn1 suggest that nonsurface chlamydial proteins could also be important for developing vaccines for *C. trachomatis*.

## 1. Introduction

Diseases caused by* Chlamydia trachomatis* (CT) infection and its sequelae represent major public health concerns with 105.7 million cases reported annually [1]. The genital tract infections caused by* C. trachomatis* have severe long-term complications and if untreated, the pathogen may ascend to the fallopian tube where it can persist for several months to years.* C. trachomatis* infection is sensitive to antibiotic treatment; however approximately 70–90% of women and 30–50% of men remain asymptomatic during infection [2]. Delayed or lack of diagnosis of* Chlamydia* is thus one of the important causes of tubal factor infertility as well as pelvic inflammatory disease (PID) [3]. Early detection and treatment would reduce the duration of infection but it may also interfere with the development of protective immune responses, resulting in increased rate of infection with reduced sequelae of chlamydial infection [4]. Therefore, understanding the mechanisms of chlamydial pathogenesis and development of effective preventive strategies are urgently needed. Characterized vaccine candidates in general have shown variable efficacy ranging from no protection to partial protection* in vivo* [5]. Both the development of protective immune responses and tissue damaging effects of infection appear to depend on the duration of infection. The first attempt to vaccinate children with whole cell vaccine resulted only in short-lived protection [6].

Experimental animal models including nonhuman primates have provided valuable information towards understanding of protective immunity to infection and testing of promising vaccine candidates. Over the years, several chlamydial antigens have been characterized; however, successful chlamydial vaccine has still not been achieved [7–9]. It has become apparent that* Chlamydia* induces both protective and pathogenic responses and hence a cautious and rational approach is required to determine safe and effective chlamydial antigens. A number of *Chlamydia-*specific antibodies have been detected during and following* C. trachomatis* infection [7, 10–12]. Antibodies recognising surface exposed epitopes of OmpA protein could neutralize chlamydial infection both in cell culture and in a mouse animal model [13–16], while antibodies against chaperones correlated with development of infections [10, 12, 17–20]. This suggests that the role of antibodies in chlamydial infection may vary depending on the antigenic epitopes recognized by immune response. Success of chlamydial vaccine development requires identification of immunogens that would be able to stimulate a protective immune response but not deleterious immune mechanisms. Most of the earlier studies correlated host immune responses to the major outer membrane protein (OmpA) and heat shock proteins (HSPs) with chlamydial protective immunity and pathogenic responses. However, neither OmpA nor HSP immune responses can account for the overall protective immunity or pathogenic responses induced during infection. These studies either focused on a few preselected antigens or were based on analysis of denatured proteins or peptides. Other membrane proteins (like polymorphic membrane proteins), cytoplasmic proteins, metabolic proteins, and secretary proteins like type three secretion system (TTSS) substrate are now being targeted as potential immunogens [8, 21, 22]. In order to fully determine the antigenic basis of host protective and pathogenic responses to chlamydial infection, an unbiased analysis of potential chlamydial antigens is required. The present study contributes to this direction by analysing the seroprevalence of potential chlamydial antigens, serine threonine protein kinase (STPK, i.e., Pkn1) and DNA ligaseA (LigA) in chlamydia-infected human patients and nonhuman primate (*Macaca nemestrina*) model of CT infection.

## 2. Methods

### 2.1. Specimen Collection

Cervical swabs and 2 mL of blood were collected from 171 patients visiting the gynaecology outpatient department of various hospitals in Delhi, India, as per the guidelines of Indian Council of Medical Research, India, and adopted by Institutional Ethical committee of Dr. B.R. Ambedkar Centre for Biomedical Research, University of Delhi (no. F50-2/Eth.Com/ACBR/11/2105), and informed consent of patients. The collected blood was allowed to clot by incubating it at room temperature for 30 minutes. The samples were centrifuged at 5500 ×g for 30 minutes. The clear sera were collected and stored at −20°C for future use. These female patients were also tested for* C. trachomatis* infection using cervical swabs and were designated as positive or negative for genital chlamydial infection using in-house PCR detection method [23, 24] and Roche amplicor MWP CT/NG Detection kit.

During the mid-1980s, Patton et al. developed an animal model using pigtailed macaques to experimentally induce chlamydial lower and upper reproductive tract disease. She studied the pathogenesis of acute and chronic chlamydial reproductive tract infections using this model. In this study, Dr. Patton provided sera from 36 infected animals and 10 uninfected control animals for use in evaluating the presence of antibodies against proteins to* Chlamydia trachomatis* [25].

### 2.2. B Cell Epitope Prediction Using BcePred Software

Bioinformatics based prediction of B cell epitopes of Pkn1, OmpA, and LigA was done by using online software BcePred, available at http://www.imtech.res.in/bic/ and developed by [26].

### 2.3. Isolation of Genomic DNA of* Chlamydia* from Clinical Samples

Clinical samples were processed according to protocol developed in the laboratory [23, 24]. To further purify DNA, phenol : chloroform extraction was performed. The aqueous phase was incubated overnight with two volumes of absolute ethanol and 1 *μ*L/mL glycogen (20 mg/mL) at −20°C. Next day, samples were centrifuged at 21,000 ×g for 30 minutes at 4°C and the pellet was washed with 70% ethanol, air-dried, and dissolved in 1X TE buffer (pH 8.0) to be used as template DNA.

### 2.4. Cloning Expression and Purification of* Pkn1*,* OmpA*, and* LigA*

All three genes* Pkn1* (1.7 kb),* OmpA* (868 bps), and* LigA *(1.9 kb) were amplified from* C. trachomatis* genomic DNA by polymerase chain reaction (PCR) using gene-specific primers:* Pkn1*: FP-GGATCCATGGACGAGCGAGCCG and RP-CACTTCGAAACCCGTAGGTACTGT;* OmpA*: FP-TTTAGAGGATCCAATGAAAAAACTC and RP-ATTATTCGAAGCGGAATTGTG;* LigA*: FP-ATCCGTGCTGTATCTCGAG and RP-CTGCAGGGAGACCGATTTTGC.

BamH1 restriction site was introduced in the forward primers of* Pkn1*,* OmpA*, and* LigA* genes. The reverse primer contained a BstI restriction site for* Pkn1* and* OmpA* while* LigA* reverse primer contained PstI restriction site. The PCR reaction mixture contained dH_2_O 35.2 *μ*L; Buffer (10X) 5 *μ*L; dNTP (2 mM) 5 *μ*L; FP (10 pm/*μ*L) 2 *μ*L; RP (10 pm/*μ*L) 2 *μ*L; template (100 ng) 0.2 *μ*L; Taq polymerase (3 U/*μ*L) 0.6 *μ*L. Reaction conditions are as follows: hot start at 94°C for 3 minutes; 36 cycles of denaturation at 94°C for 45 seconds; annealing 60°C for 45 second extension at 72°C for 45 seconds. The desired PCR products were cloned in pGEM-T easy vector (Promega) and subsequently cloned in pTrcHisC expression vector (Invitrogen) and confirmed by sequencing. Single colony of each* Pkn1*,* OmpA*, and* LigA* transformed in XL1/BL-21 strain of* E. coli* was used for overexpression in liquid culture by inducing with 0.4 mM IPTG for 3.5 hours at 30°C on shaker incubator. Purification was carried out using Ni-NTA affinity chromatography. The collected fractions were analysed on SDS-PAGE using Coomassie brilliant blue as well as western blot.

### 2.5. ELISA of the Purified Fusion Proteins

The indirect ELISA approach was used to determine the IgG response to Pkn1, OmpA, and LigA proteins in sera samples. For the sera samples obtained from* M. nemestrina*, HRP conjugated anti-monkey IgG (Fitzgerald, USA) and for human patient sera anti-human IgG (Santacruz Biotechnology, USA) were used as secondary antibodies. Standard ELISA method was followed. The antigen was diluted in carbonate buffer (pH 9.5) to a final concentration of 10 ng/*μ*L and 50 *μ*L of the latter was seeded per well of the 96-well ELISA plate followed by overnight incubation at 4°C. The plate was washed with PBS-Tween20 (PBST), 3-4 times. Thereafter blocking solution (1% BSA) was added and incubated for 2 hours at 37°C, followed by washing with PBST 3-4 times. Diluted sera samples were incubated for 1.5 hours at 37°C then washed (3-4 times) with PBST. Secondary HRP conjugated antibody was added and incubated for 1 hour at 37°C, followed by washing of the plate with PBST 3-4 times and developed using 3,3′,5,5′-Tetramethylbenzidine (TMB) as substrate. The reaction was terminated using 2 N HCl. Each sample was tested in triplicates and the absorbance at 450 nm was measured (*A*_450_) and mean value of* A*_450_ for each sample was calculated as a measure for the seroreactivity of Pkn1, OmpA, or LigA. The cutoff value was decided by the formula: Cut off = Mean ± (3X S.D.). Mean is the average of all the healthy samples considered and S.D. is the standard deviation of the healthy samples. From the cutoff value it was decided whether the sample was positive (values greater than cutoff) or negative (values lower than the cutoff) for antibodies against Pkn1, OmpA, and LigA.

### 2.6. Statistical Analysis

All statistical analyses were performed using Graph-Pad Prism version 5 (Graph-Pad Software Inc., San Diego, CA, USA). The results of ELISA assays are presented as mean ± SD. Spearman's rank method was used to find any correlation between antichlamydial antigens.

## 3. Results

### 3.1. B Cell Epitope Prediction for Pkn1, OmpA, and LigA

To initiate our study, bioinformatic analysis of full length Pkn1 and LigA protein sequences was performed to identify antigenic stretches for B cells epitopes using BCePred software (Table 1). Similar analysis was also performed for the well-established chlamydial antigen OmpA that has been used as a positive control throughout the study.

Upon careful analysis we observed that in Pkn1 and OmpA top scoring epitopes were present towards C-terminal and N-terminal, respectively, while top scoring epitopes in LigA were distributed in middle region. Considering that N- and C-terminal regions of proteins are usually solvent accessible, the possibility of antibodies response against these regions is much higher [27].

### 3.2. Cloning Expression and Purification of Pkn1, OmpA, and LigA

**Figure 1:**
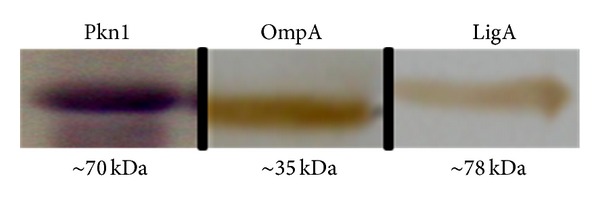
Western blot showing presence and purity of His tagged proteins, Pkn1, OmpA, and LigA.

All three recombinant proteins (Pkn1, OmpA, and LigA) were solubilized and purified using Ni^+^-NTA chromatography. Pkn1 and OmpA were eluted at 300 mM imidazole while 100 mM imidazole was used to elute LigA (Figure S1 in Supplementary Material available online at http://dx.doi.org/10.1155/2014/245483). Purity of the proteins was checked by Coomassie staining of SDS-PAGE while the identity of the recombinant proteins was confirmed by western blot analysis using anti-his antibodies (Figure 1).

The purified proteins were tested to determine IgG antibody response to Pkn1 and LigA in serum samples of* M. nemestrina* and human patients infected with* C. trachomatis*. The antibody titres were measured using antigen-specific ELISA. Since antibodies to OmpA are typically elicited in response to chlamydial infection [28–30], IgG levels against OmpA were measured by an OmpA-specific ELISA as a positive control and to compare the relative immune response elicited towards Pkn1 and LigA in the sera of* M. nemestrina* and human patients.

### 3.3. Detection of Antibodies against Pkn1, OmpA, and LigA in Sera of* M. nemestrina* Infected with* C. trachomatis*

Serum samples (*n* = 46) from* M. nemestrina *(pigtailed macaques), an animal model of experimentally induced* C. trachomatis* infection [25], were used to test for the presence of antibodies against Pkn1, OmpA, and LigA. All 46 sera samples were tested for seroreactivity with the three proteins (Table 2). The ELISA results of the uninfected control sera (*n* = 10) were used to decide the cutoff value as described in Methods.

**Figure 2:**
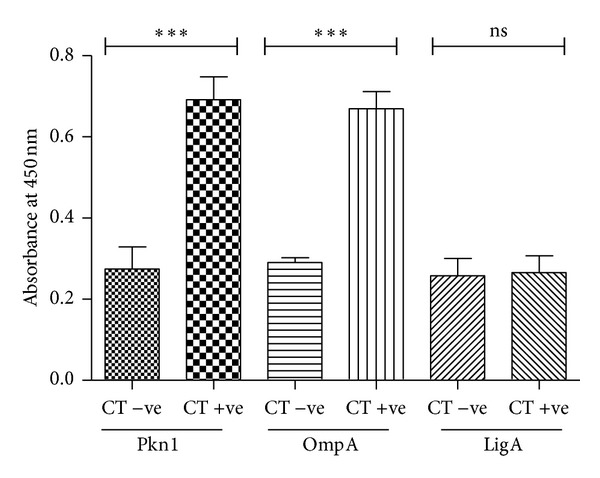
Quantitation of chlamydial protein-specific antibodies in sera of* M. nemestrina* by ELISA using recombinant chlamydial proteins. Mean values of seroreactivity against Pkn1, LigA, and OmpA were measured in sera of* C. trachomatis* infected (CT +ve) and uninfected (CT –ve)* M. nemestrina*. ***Represents *P* < 0.001, that is, highly significant. **Represents *P* < 0.01. *Y* axis is the absorbance of antichlamydial antibodies measured at 450 nm; *X* axis is the chlamydial antigens in CT +ve and CT −ve sera.

Prevalence of anti-OmpA and anti-Pkn1 antibodies was significantly higher (*P* < 0.001) in sera of chlamydia-infected animals than in sera from uninfected animals (*A*_450_ mean values: 0.67 v/s 0.29 and 0.69 v/s 0.27, respectively; Figure 2). Seropositivity to OmpA was observed in 78% of the sera samples (28/36, *χ* ^2^, *P* < 0.001) from* C. trachomatis*-infected* M. nemestrina*. For Pkn1 67% (24/36, *χ* ^2^, *P* < 0.001) of these sera showed positive reactivity. On the other hand, no significant seroreactivity was observed with LigA. Our results suggest that the serum of* M. nemestrina* infected with* C. trachomatis* show significant seroprevalence of antibodies against chlamydial recombinant proteins Pkn1 and OmpA but not LigA. It is pertinent to mention that since* M. nemestrina* is not a natural host of* C. trachomatis*, it was important to establish the immune response elicited by Pkn1 in humans. Therefore, we tested the sera of human patients (uninfected and infected) for the presence of antibodies against Pkn1 and LigA.

### 3.4. Detection of Antibodies against Pkn1 and OmpA in Human Sera from Patients Infected with* C. trachomatis*

**Figure 3:**
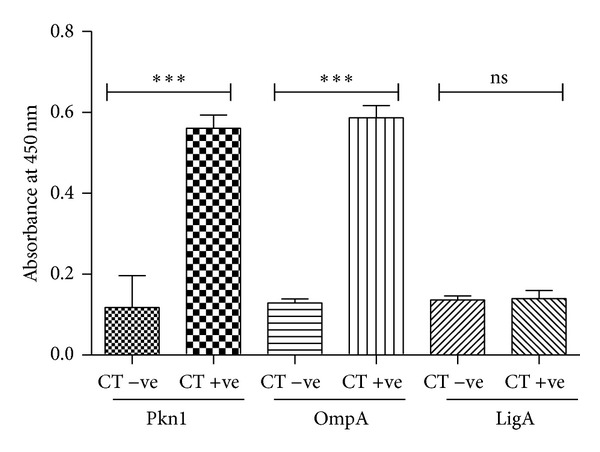
Quantitation of chlamydial protein-specific antibodies in sera of human patients uninfected and infected with* C. trachomatis* by ELISA using recombinant chlamydial proteins. Mean values of antibodies against Pkn1, OmpA, and LigA were measured in sera from* C. trachomatis* positive (CT +ve) and negative (CT –ve) sera. ***Represents *P* < 0.001, that is, highly significant. **Represents *P* < 0.01. *Y* axis is the absorbance at 450 nm (*A*_450_) of antichlamydial antibodies measured at 450 nm; *X* axis is the chlamydial antigens in CT +ve and CT –ve sera.

Natural human antibody response to Pkn1 and LigA following chlamydial infection is not known. Sera samples (*n* = 171) were collected from patients as described previously (under materials and methods). The endocervical swabs were also collected from these patients for testing the status of infection with* C. trachomatis*. Thirty patients tested positive for chlamydial infection while 141 were uninfected based on results of in-house PCR and Roche Amplicor MWP kit. All 171 sera samples were tested for anti-Pkn1, anti-OmpA, and anti-LigA antibodies (Table 3). ELISA results for anti-OmpA and anti-Pkn1 antibodies in patient's sera showed significantly higher* A*_450_ values in* C. trachomatis* positive patients than in* C. trachomatis* negative patients (mean values: 0.5867 v/s 0.1288 and 0.5609 v/s 0.1175, resp.; (*P* < 0.001), Figure 3). All samples positive for anti-OmpA and anti-Pkn1 antibodies had* A*_450_ values greater than cutoff value. When tested for anti-LigA, once again we did not observe any seroreactivity even when used at 10 times higher concentrations than that of Pkn1.

Antibodies to OmpA were present in 93% (28/30, *χ* ^2^, *P* < 0.001) of the sera from* C. trachomatis*-positive women and 87% (26/30; *χ* ^2^, *P* < 0.001) of sera showed reactivity to Pkn1 (Table 3). Sera from 134 women (out of 141), testing negative for* C. trachomatis* infection, showed no reactivity to OmpA or Pkn1 (Table 3). When the reactivity within the* C. trachomatis* positive group was compared, antibody levels against OmpA (mean* A*_450_ = 0.58) were not significantly different (*P* > 0.05) from those of Pkn1 (mean* A*_450_ = 0.56) (Figure 3). These results suggest that following a natural* C. trachomatis* infection in humans, antibody responses to Pkn1 are comparable to those of OmpA. Similar experiments performed by Kawa et al. [30] with chlamydial PorB protein did not elicit strong antibody response in comparison to OmpA. A consistent antibody response against Pkn1 observed during chlamydial infection in pigtailed macaques and in humans raises the possibility of Pkn1 as a candidate immunogen.

**Figure 4:**
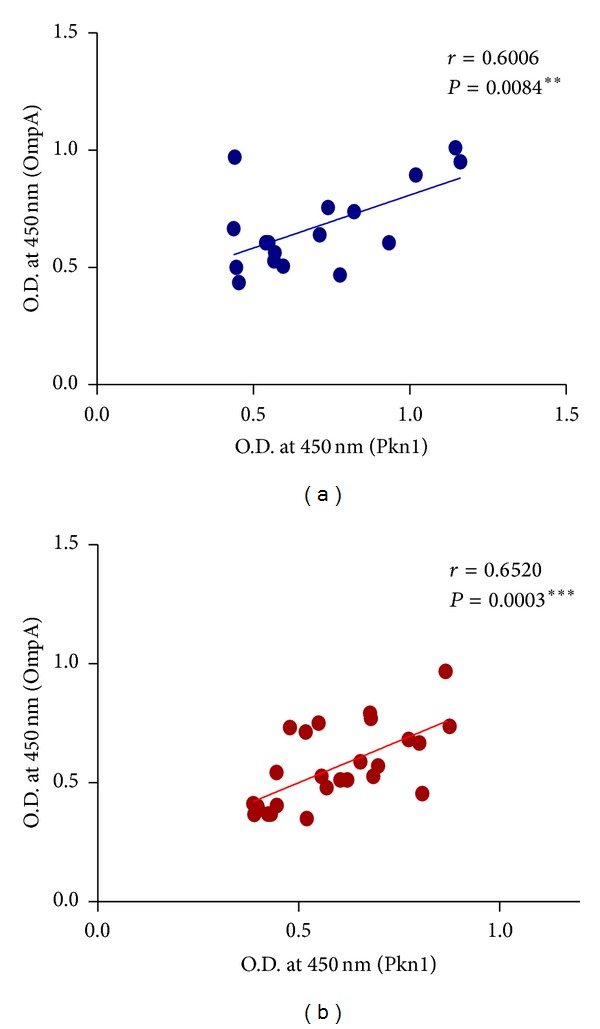
Correlation of antibody titres against OmpA and Pkn1 in CT positive sera of* C. trachomatis* infected* M. nemestrina* (a) and human patients (b).

Since OmpA is known to induce an immune response in patients infected with* C. trachomatis* [29, 31] we looked at the correlation between antibody titres against OmpA and Pkn1. A significant positive correlation between OmpA and Pkn1 was observed (*r* = 0.6006 for macaques and *r* = 0.6520 for humans; Figure 4) that further supports our hypothesis that Pkn1 could be an important immunogen.

**Figure 5:**
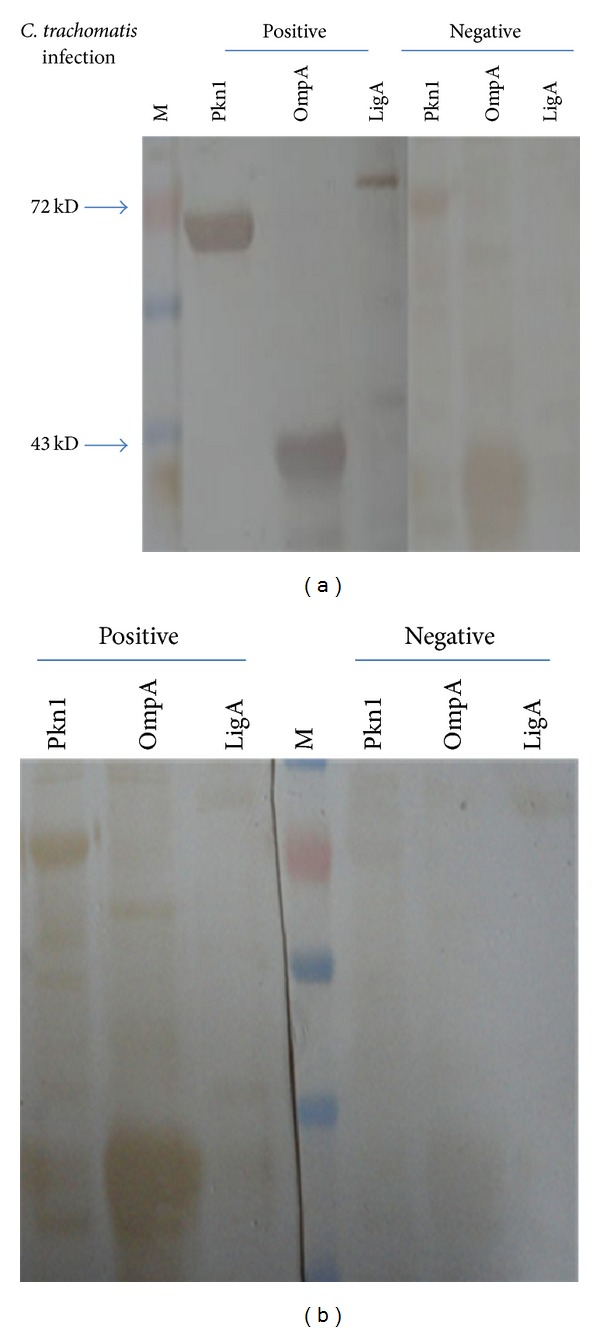
Western blot using pooled sera from* Chlamydia* infected samples of human and macaques (*M. nemestrina*) shows reactivity with purified recombinant proteins Pkn1, OmpA. As evident, sera from infected patients (a) and macaques (b) reacted with purified Pkn1 and OmpA but react poorly with LigA protein. The sera from uninfected patients do not react with any of the three antigens.

To further support our observations of a significant seroprevalence of anti-Pkn1 antibodies in chlamydial infections, the purified chlamydial proteins were immunoblotted using pooled sera samples as shown in Figure 5. Sera pooled from CT-infected* M. nemestrina* and human patients detected Pkn1 and OmpA (~70 kDa and ~35 kDa, resp.). For LigA, a band of the correct molecular weight (~78 kDa) was observed with pooled sera from CT-infected patients; however the intensity of the band was significantly weak in comparison to the bands observed for Pkn1 and OmpA. Although this result can indicate low seroprevalence of anti-LigA antibodies, it does not corroborate LigA to be a candidate immunogen in CT infection since when the samples were tested individually in ELISA, no significant immune response was observed in CT-positive sera of human (Figure 3 and Table 3). In addition insignificant difference was seen between western blots for LigA using pooled sera from CT positive and CT negative* M. nemestrina*. In summary, among the two chlamydial proteins tested (Pkn1 and LigA), our results conclusively demonstrate significant seroprevalence of antibodies against Pkn1 in* C. trachomatis* infection.

## 4. Discussion

The use of an effective vaccine appears to be the most viable option for the long-term control of chlamydial infections [32–34]. Such a vaccine must be capable of eliciting both humoral and cellular immune responses that are required for protection and elimination of chlamydial infections. However, vaccine development for* C. trachomatis* remains challenging partly due to the potential pathologic effects of the host's immune response to* Chlamydia* [35]. Vaccine development for* C trachomatis* was mostly focused on OmpA and structural proteins. Antigenically variant OmpA confers serovar-specificity to chlamydial isolates [29, 31] and restricts antibody-mediated neutralization. The polymorphism seen in OmpA has been attributed to immune selection pressure rather than differences in biological function suggesting that* C. trachomatis* employs antigenic variation as a strategy for immune evasion [36]. Furthermore, OmpA-mediated antibody neutralization is conformation dependent [15]; as a result, vaccine testing and development using OmpA derivatives have been particularly difficult. It has become a research priority to identify alternatives to OmpA that may have promise for eliciting protective immune responses. OMP2 is more highly conserved in amino-acid sequence among different* C. trachomatis *serovars than OmpA [37] and hence may be better suited as a vaccine candidate. Other vaccine candidates are Hsp60 isoforms as their prolonged exposure leads to immune system activation and antibody formation [38]. Some Pmps, including Pmp-B, -C, -D, and -I, may be more abundantly expressed or specifically exposed at the chlamydial surface to elicit a relatively stronger antibody response. These proteins are capable of eliciting quantitatively and qualitatively different antibody responses in patients [39]. It has also been realized that both cell mediated and humoral immune response will be necessary for a successful chlamydia vaccine and both native and surface conformation will constitute an important feature of a particular chlamydial antigen [9]. However, to date no successful vaccine or effective component immunogens have been characterized.

Earlier studies have also suggested that nonsurface chlamydial proteins that are less abundant than OmpA can be potent immunogens [28, 40]. Recent studies also showed that surface and cytoplasmic proteins play equally important roles during development of immunity against* Chlamydia* [8, 22]. The purpose of this study was to characterize the natural human immune response to the chlamydial proteins Pkn1 and LigA. Pkn1 is a soluble, nonsurface chlamydial protein. Studies by [41] indicated that Pkn1 interacts with IncG, a well-established TTSS substrate. Studies in* Yersinia pestis* showed that YpkA, a protein similar to STPK, is injected into the host cell via yersinial TTSS. These observations suggested that Pkn1 might also be secreted through TTSS. It has been implicated that proteins involved in TTSS could be potential candidate immunogens [4, 42–44]. Since TTSS effectors include proteins with kinase activity, it led to the speculation that Pkn1 may be TTSS substrate and could serve as potential immunogen [4]. The prospects of nonsurface chlamydial proteins as promising vaccine candidates prompted us to explore the immunogenic potential of Pkn1 and LigA.

This study clearly demonstrates significant seroprevalence of anti-Pkn1 IgG in humans infected with* Chlamydia* as well as in experimentally induced CT infected* M. nemestrina*. Analysis of sera from 171 samples of women out of which 30 were diagnosed with a* C. trachomatis* infection revealed presence of antibodies against Pkn1 at a level comparable to that of well-established immunogenic protein OmpA. On the other hand no significant seroreactivity was observed for LigA. One possible reason could be that for both OmpA and Pkn1 the top scoring epitopes predicted using BcePred software were localized in the N or C-termini, whereas for LigA these epitopes were enriched in the solvent inaccessible middle region (Table 1). Identification of immunodominant proteins like Pkn1 emphasizes the role of nonsurface proteins in modulation of the host immune response in chlamydial infection and may in combination with chlamydial surface proteins play important role in the vaccine development programmes for* C. trachomatis. *Success of chlamydial vaccine development, however, requires identification of immunogens that would be able to stimulate a protective immune response but not deleterious immune mechanisms.

## Supplementary Material

Supplementary Figure 1: Chlamydial antigens LigA, Pkn1 and OmpA were purified using Ni-NTA affinity chromatography and were analysed on SDS-PAGE gel using Coomassie brilliant blue. Respective antigens and their molecular weights are shown.

## Figures and Tables

**Table:** (a) Pkn1

Rank	Sequences	Start position	Score
1	SLKEDLRCAHRHRNNP	583	0.94
2	YEWCQDWYSYDFYENS	543	0.93
2	FFSSDTTPVMSYPANI	517	0.93
3	DFYENSALEPDSPQGP	553	0.90
4	PGAINSTYGFRCAKDV	598	0.89
4	YGASSYASWIGKRLPS	472	0.89
5	YLLVGAFPWGAFPKPS	215	0.88
6	GGKLGMRYPTGEDVDK	497	0.87
7	IIEPGYAKHPVVGVTW	456	0.86

**Table:** (b) OmpA

Rank	Sequences	Start position	Score
1	EGFGGDPCDPCATWCD	41	0.95
2	CATWCDAISMRVGYYG	51	0.92
3	TGTKDASIDYHEWQAS	259	0.91
4	YHEWQASLALSYRLNM	268	0.90
5	TGNSAAPSTLTARENP	92	0.89
6	IAVGTTIVDADKYAVT	359	0.87
7	GRHMQDAEMFTNAACM	110	0.87
8	GAEGQLGDTMQIVSLQ	332	0.86

**Table:** (c) LigA

Rank	Sequences	Start position	Score
1	CQREQGKLEFANPRNA	178	0.94
2	TELVEHDRRYYVLNQP	7	0.92
3	CSDIFALAEEDLKQVP	465	0.92
4	VERIREIEEMRAALPM	255	0.91
5	QVGKTGILTPVAELAP	318	0.90
6	DRSIQNLLASIAGAKK	475	0.88
7	SEPWKMPSLCPVCHEP	387	0.88
8	MRLPQEAPEDLEVRGE	149	0.88

**Table 2:** ELISA results for the seroprevalence of anti-Pkn1, anti-OmpA and anti-LigA antibodies in sera of *M. nemestrina* (Macaque model) infected with *C. trachomatis* and control group.

Number of sample	Immune response as determined by ELISA			Chlamydial infection∗
	Pkn1	OmpA	LigA	
18	+	+	−	+
10	−	+	−	+
6	+	−	−	+
2	−	−	−	+
10	−	−	−	−

Diluted sera of *M. nemestrina *were incubated with respective antigens followed by HRP conjugated secondary antibodies using standard protocol as described under methods. *A*_450 _ above the cut off value was considered as positive samples; the cut-off value for OmpA = 0.40 while cut-off value for Pkn1and LigA was 0.43 and 0.39 respectively. ∗The status of chlamydial infection was as provided by the laboratory of Prof. Dorothy L. Patton, University of Washington, Seattle, WA, U.S.A.

**Table 3:** ELISA results for the seroprevalence of anti-Pkn1 and anti-OmpA antibodies in sera of Chlamydia-infected and uninfected patients.

Number of samples	Immune response as determined by ELISA			Chlamydial infection∗
	Pkn1	OmpA	LigA	
26	+	+	−	+
134	−	−	−	−
4	−	+	−	−
2	−	−	−	+
3	+	−	−	−
2	+	−	−	+

100 ng each of the purified protein was used for ELISA. The cut off value for Pkn1: 0.34, OmpA: 0.35 and LigA: 0.34. Absorbance (*A*_450_) above the cut off value was considered as positive samples. ∗The in-house PCR assay and Roche MWP kit results were used as the basis to decide the presence or absence of chlamydial infection.
