# Supplementary material for: Seroprevalence of Antibodies against Pkn1, a Novel Potential Immunogen, in Chlamydia trachomatis-Infected Macaca nemestrina and Human Patients
Source: Biomed Res Int. 2014 Jun 18;2014:245483. doi: 10.1155/2014/245483 (PMC4086347; doi:10.1155/2014/245483)
Supplement: Supplementary file 1 — Supplementary Figure 1: Chlamydial antigens LigA, Pkn1 and OmpA were purified using Ni-NTA affinity chromatography and were analysed on SDS-PAGE gel using Coomassie brilliant blue. Respective antigens and their molecular weights are shown. [file 245483.f1.ppt]

## Slide 1
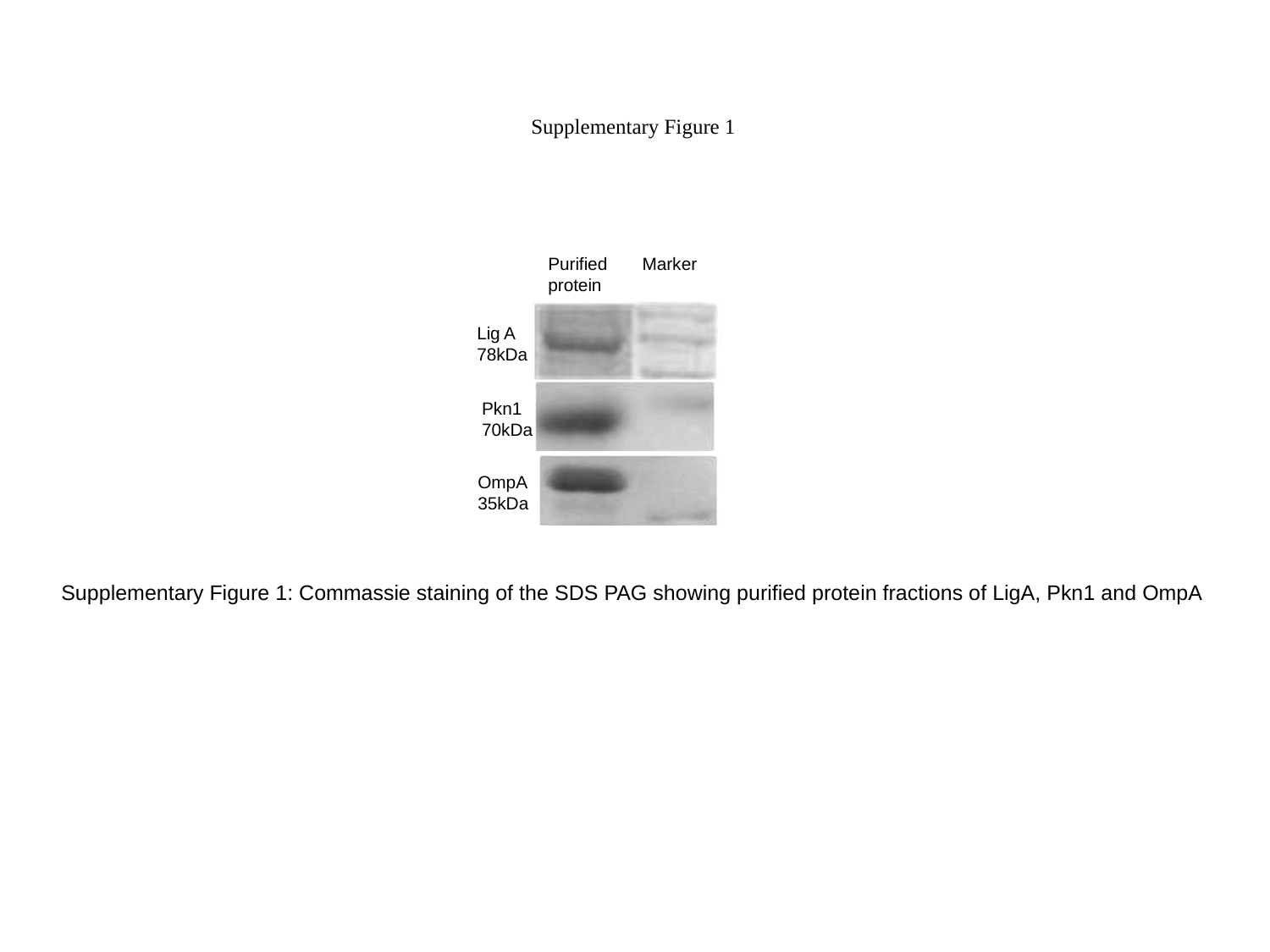

Supplementary Figure 1
Purified
protein
Marker
Lig A
78kDa
Pkn1
70kDa
OmpA
35kDa
Supplementary Figure 1: Commassie staining of the SDS PAG showing purified protein fractions of LigA, Pkn1 and OmpA
